# Robust Identification of Polyethylene Terephthalate (PET) Plastics through Bayesian Decision

**DOI:** 10.1371/journal.pone.0114518

**Published:** 2014-12-08

**Authors:** Mohd Asyraf Zulkifley, Mohd Marzuki Mustafa, Aini Hussain, Aouache Mustapha, Suzaimah Ramli

**Affiliations:** 1 Department of Electrical, Electronic and Systems Engineering, Faculty of Engineering and Built Environment, Universiti Kebangsaan Malaysia, 43600, Bangi, Selangor, Malaysia; 2 Department of Computer Science, Faculty of Defence Science and Technology, Universiti Pertahanan Nasional Malaysia, Kem Sungai Besi, 57000, Kuala Lumpur, Malaysia; Southwest University, China

## Abstract

Recycling is one of the most efficient methods for environmental friendly waste management. Among municipal wastes, plastics are the most common material that can be easily recycled and polyethylene terephthalate (PET) is one of its major types. PET material is used in consumer goods packaging such as drinking bottles, toiletry containers, food packaging and many more. Usually, a recycling process is tailored to a specific material for optimal purification and decontamination to obtain high grade recyclable material. The quantity and quality of the sorting process are limited by the capacity of human workers that suffer from fatigue and boredom. Several automated sorting systems have been proposed in the literature that include using chemical, proximity and vision sensors. The main advantages of vision based sensors are its environmentally friendly approach, non-intrusive detection and capability of high throughput. However, the existing methods rely heavily on deterministic approaches that make them less accurate as the variations in PET plastic waste appearance are too high. We proposed a probabilistic approach of modeling the PET material by analyzing the reflection region and its surrounding. Three parameters are modeled by Gaussian and exponential distributions: color, size and distance of the reflection region. The final classification is made through a supervised training method of likelihood ratio test. The main novelty of the proposed method is the probabilistic approach in integrating various PET material signatures that are contaminated by stains under constant lighting changes. The system is evaluated by using four performance metrics: precision, recall, accuracy and error. Our system performed the best in all evaluation metrics compared to the benchmark methods. The system can be further improved by fusing all neighborhood information in decision making and by implementing the system in a graphics processing unit for faster processing speed.

## Introduction

Efficient waste management has become a major concern throughout the world in both the developed and developing countries. The European Environment Agency has projected that total municipal waste produced by the European countries will increase by 25% in 2020 as compared to 2005 [1] and in Beijing alone, 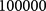 tonnes of PET has been consumed in 2012 [2]. There are three most common methods to treat municipal waste: recycling, incineration and landfill. Landfill is the most popular method where the waste will be buried inside the earth. However, not all waste can be disintegrated easily by the nature, especially plastics materials that may take more than 100 years to be biodegradable. Hence, recycling has become the second most preferred waste treatment where the waste will be processed and reused again.

The first step towards recycling is sorting the waste to classify it according to its type. Different types of waste require different methods of treatment. Usually, there will be a dedicated waste bin for the popular types of recycle waste such as paper, plastic and glass. Even for plastic waste, they will be various methods of treatment that require them to be discriminated, mainly according to the material type. The highest scrap value among the plastic waste is polyethylene terephthalate (PET) because it is environmentally friendly and low cost [3]. PET plastic can be easily recycled where it will be shredded into flakes before purification and decontamination processes are performed. Quality of the recycled PET depends heavily on how well the waste can be separated and purified. Thus, the sorting process is a very important step in improving the quality of the recycled materials.

However, recycling processes for the PET material do pose some health concerns, where there is a possibility of antimony leaching into the PET material as reported by Takahashi et al. [4]. The authors claimed that the quantity of antimony leaching is within an acceptable amount but the long term effect of continuous recycling of PET should be analyzed for possible health effects. Due to popular demand for PET material that is used in beverage packaging, medium and large recycling centers have implemented automated systems to classify plastic wastes mainly into PET and non-PET materials. Small scale operations will usually sort the waste manually, which requires a lot of effort and time. The quality of sorting also will degrade once the workers feel tired. Apart from manual sorting, some recycling centers have adopted chemical based systems to identify the plastic material as used in [5]. The main disadvantage of this method is the residue from the chemical waste. The danger of inefficient chemical waste management may overcome the benefit of recycling the plastic waste.

Electrostatic approach, Park et al. [6] has also been employed to sort the plastic waste, in which the materials are cut to a small size before being charged and deflected to respective nodes based on the material type. Generally, medium and small scale operations cannot afford such a complicated system because of the expensive start up cost of the sorter mechanism and high maintenance cost. Hence, mechanical based systems have been employed to develop a safe approach to plastics sorting such as the system in [7]. Vision based sensors are the most versatile method in identifying the PET material, where it is identified, so that a hydraulic or pneumatic mechanism can be instructed to direct the material to the right waste bin. The main advantages of vision based systems are it just requires a simple construction, cheap, fast and non-intrusive approach. Charge-coupled device (CCD) cameras are the most frequently employed sensors in vision based PET plastics sorters.

Our proposed method is based on a probabilistic approach to identify the opaqueness of the reflection points and their correlation to the neighboring regions. Our system works by assuming that the neighboring regions around the reflection points for the PET bottle will most likely be a transparent region. We introduce a white strip approach in which the reflection point will be combined together to form a slender strip. A collection of reflection points will form a white edge as captured by the CCD camera. This white edge can be the real reflection points as well as the white region that may originate from the bottle label. Thus, the slender shape assumption will be crucial in detecting the true reflection points. The main novelty of our methodology is the probabilistic modeling of the white strips and their neighboring regions. The advantage of the probabilistic decision can be observed through better sorting accuracy, especially for imperfect bottle conditions due to dents, labels and dirt. The neighboring regions of the white strip are analyzed to assign the probability that the white strip may belong to a PET bottle or not. Since there will be many white strips detected for a bottle, the statistical data is collected in histogram form that will be weighted by the white strip size. The decision also will be made probabilistically by a likelihood test before a pneumatic mechanism will direct the bottle to the right bin.

This paper is organized into 5 sections. Section 2 is a literature review that covers various methods of PET material detection. Section 3 on methodology is an in depth explanation of the whole system such that more emphasis will be given to the detection scheme. Extensive results and discussion are presented in section 4 that validates how well the probabilistic white strip approach works as compared to other existing camera based PET detection systems. A concise conclusion and several recommendations will be shared in the last section 5.

## Literature Review

Generally, there are three forms of PET monomer produced for the industrial usage, which are fiber, bottle and film. Bottle grade PET accounted for 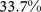 of total PET produced in 2010 [8]. There are four general categories of recycling methods according to Nikles et al. [9] where the primary recycling method deals with uncontaminated waste while the secondary recycling method uses mechanical processes to remove the contaminants. The third recycling method employs chemical treatment that transforms the polymer chain of waste into a monomer. Lastly, the fourth recycling approach is incineration in which the waste is burned in a combustion chamber.

This paper concerns only the second recycling method that requires separation of the plastics waste based on its respective material to produce a high quality recycling material. The most popular type of sensor used in industrial applications to sort the waste is a near infrared (NIR) sensor as used in [10] where the reflective wavelength of the waste bottle is used to determine its type. Its main disadvantage is the overlapping range of the reflective properties of some materials. Apart from that, it does not factor in the size and color of the waste, which is crucial in differentiating waste that has been contaminated.

NIR sensors have been employed in plastics identification systems since 1998 [11]. They classified household wastes automatically into two classes either plastics or non-plastics material. A database of spectroscopic images of wastes is built beforehand as the basis for the material comparison. Their approach is sensitive to temperature and humidity variations. Artificial neural networks method is used to classify the wastes where the parameter weights are determined through supervised training for both plastics and non plastics cases to produce optimal recognition.

Instead of using the spectroscopic image directly, Barcala et al. [12] used wavelet transformation to reduce the information size of the captured data. They used a fast NIR detector through acousto-optic tunable filter technology. Wavelet transformation is able to reduce the spatial and flickering noise. Wavelet representation is then used to set up a coefficient set, which is the foundation for quaternion number generation. Similar to before, a database of quaternion numbers for domestic waste is constructed where a plastic waste is recognized if its number matched with any of the database number.

Rather than relying solely on NIR sensor, Tachawali et al. [13] fused a charge-coupled device (CCD) camera and NIR in a two-stage identification process. The first stage relies on NIR, which detects two global minimum dips in spectroscopy history. Different materials will have different dip amplitudes that occur at unique frequencies. The second stage used a CCD camera that captures the image in RGB format which is then converted to HSI space. The bottle was first aligned using principal component analysis for better segmentation. Five rows are created at the bottom and top regions of each bottle where mean and standard deviation values of hue and saturation channels are extracted as the features. The system classified the bottle into three categories of clear, natural and opaque plastics through a fusion of quadratic and tree classifiers. The computational load of the system is heavy even though the authors claimed that their system can work in real time.

As previously mentioned, a NIR sensor alone is not sufficient to produce a high accuracy and precision system. It lacks spatial information to recognize the shape of the plastics bottle as well as color information that can be used to distinguish between the label and the plastic regions of plastic waste. Hence, a visible spectroscopic camera is used by House et al. [14] to sort the plastics waste in real time. Their method assumes that there is no overlapping and touching between the plastics wastes. A background subtraction method is employed to locate the bottles where their region will be cropped for building the histogram. A region growth algorithm is used to maximize the size of the detected foreground and to fill in any gap. Grey scale histogram of the bottle is then fed into support vector machine (SVM) for classification. SVM has been proven by [15] to be able to distinguish between PET and non-PET materials. The algorithm by House et al. can perform a real time operation but the simple feature of grey scale signature is not enough when the lighting condition is not stable. Any slight variation in the illumination will reduce the accuracy of detection and the SVM will require retraining to derive a new hyperplane.

Scavino et al. [16] overcame the problem of touching through genetic algorithm (GA). They still assumed no overlapping between the wastes, but touching incident may occur and it is hard to separate them if the wastes pattern is similar. GA is used to derive the optimal straight line that separates the bottles. The initial population of the lines is determined heuristically, which will be combined and mutated before a final fit is found. The performance of this approach will degrade when the bottles cannot be separated by a straight line, especially when the bottle is crushed and deformed. Moreover, there is also the case where more than two bottles touch together, which makes separation by a single straight line impossible.

Apart from normal visible light analysis, Picon et al. [7] have used a hyperspectral image that extends the visible light wavelength to also include the NIR wavelength. They showed that raw materials can be segregated based on their unique spectral-spatial feature, which they proposed as a fuzzy spectral and spatial classifier (FUSSER). However, their approach is less accurate if the contaminant or label obstructs the waste image so that the captured image represents a mix composition of materials.

To overcome these challenges, Safavi et al. [17] used a visible reflectance spectroscopy image to identify various types of plastics from municipal solid waste. Their algorithm is based on color feature, which is focused on three additive primary colors. The algorithm mitigates the label and contamination issues by selectively identifying the right region and limiting the maximum size of the label. Sequential tests starting from the highest to the lowest reflectance wavelength are used to identify the color of the plastics, which should be coherent unlike a label. However, they admit that their method is not suitable if the label noise is too high as they have employed a rigid thresholding method.

Artificial neural networks (ANN) has also been used to classify PET and non-PET bottles. [18] utilized structuring elements as the input to the ANN classifier, which are the vertical, horizontal and 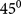 lines. Their assumption is a PET bottle will have a more slender shape as opposed to non-PET bottles that are relatively square in shape. This assumption is not generally accurate as some of the non-PET bottles do have slender shapes.

Instead of relying on physical shape information alone, Zulkifley et al. [19] introduced a probabilistic white strips approach. A PET bottle is recognized through neighborhood information of the reflection area, which is usually white in color. However, not all white regions are the reflection area, such as a white label or white contaminant. Thus, a physical feature has been fused together with color information to decide the reflection regions. Neighborhood data is then used to infer the material type through comparison of the transmittance between the reflection region and its surrounding areas.

## Methodology

**Figure 1:**
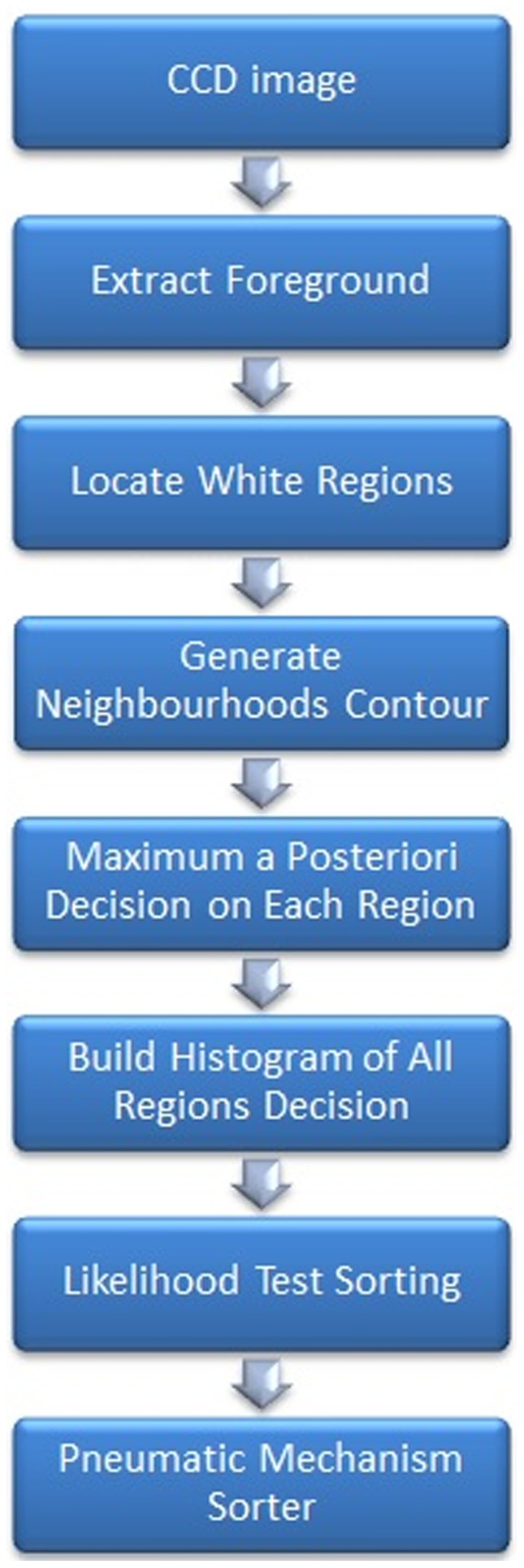
Process flow of the complete sorting system.

The first step to sort the wastes is to identify and classify them based on the materials. Our material identification is based on the computer vision technique, which derives the material type through transmittance characteristics of the neighborhood regions of the reflection strip. We define this strip as the “white strip”, which is the reflection area of the bottle as seen from the camera point of view. It is a good feature to distinguish either the white color is originated from the label or the plastic itself. A true bending region that corresponds to the plastic will have a higher probability to be slender in shape before a mechanical sorter such as pneumatic ejection will put the waste into the right sorting bin. The complete flow of the sorting process is illustrated in Fig. 1.

**Figure 2:**
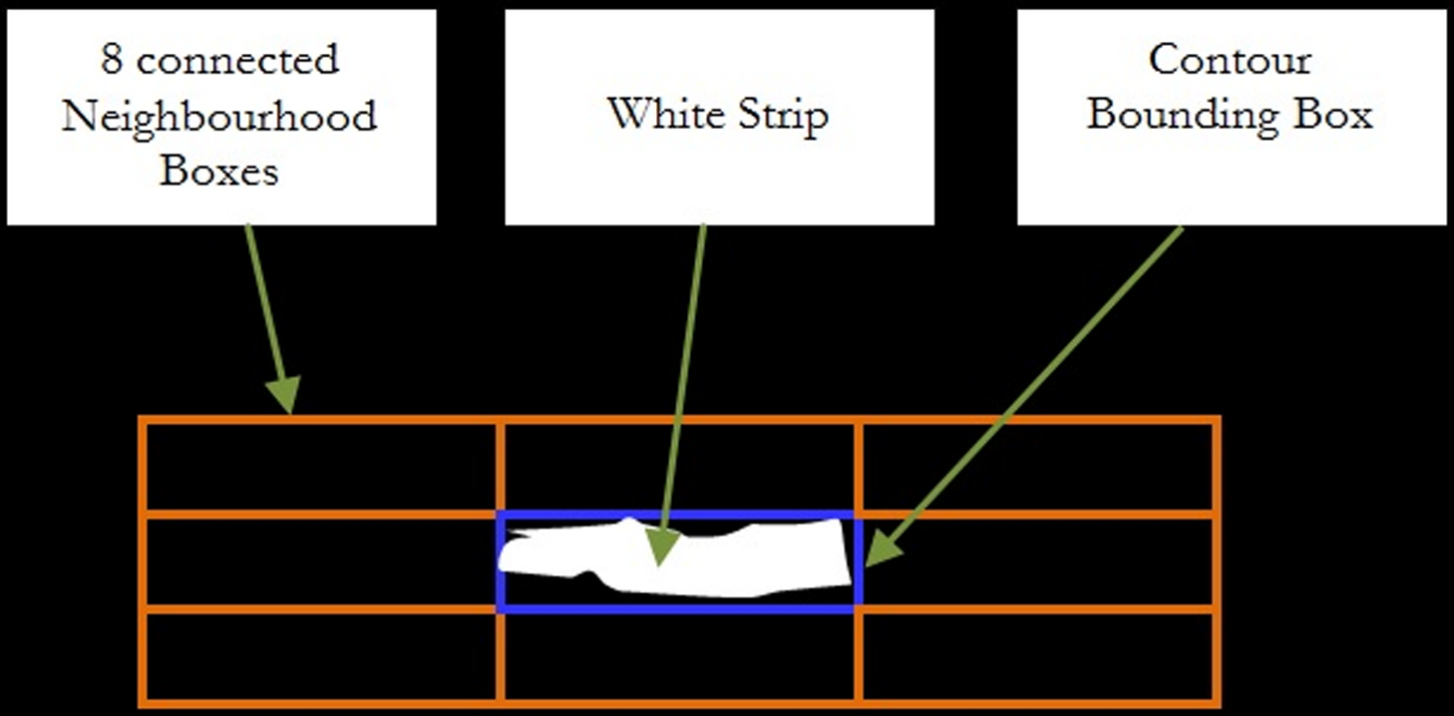
An example of 8 neighbourhood boxes built around a white strip.

The neighborhood areas of the white strip are divided into 8 equal boxes that are the same size of the white strip. These 8 boxes are called the neighborhood boxes or grey strips as shown in Fig. 2, in which the output decision of the highest probability grey strip between them will be accumulated in histogram form. There are three parameters used in modeling the neighborhood information of the white strip to distinguish between a PET and non-PET material. These are the centroid distance between grey and white strips, ratio of height over width of the grey strip and color information of each individual pixel in the grey strip.

**Figure 3:**
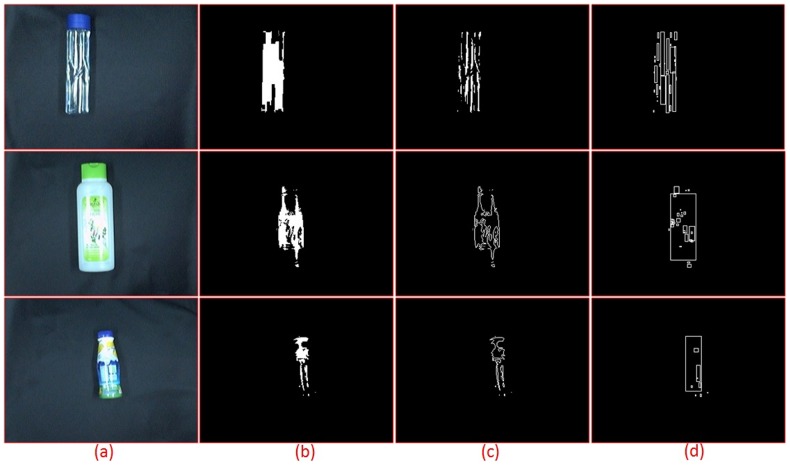
Sample of processed wastes: (a) input waste (b) Foreground image (c) True white strips (d) Bounding boxes of the contours.

The process starts by capturing the waste images by using a CCD camera that is mounted on top of the conveyor belt to get a clear zenith view. A foreground extraction method is employed to extract the waste image from the conveyor. Initially, the conveyor belt image is captured before any waste is processed, which will become the background model and the basis for the background subtraction method. An 8-connected pixel approach is used to link up each of the individual detected foregrounds to group them together as well as to remove the noise. Erosion and dilation filters are applied to further smooth out the boundary pixels. The minimum number of connected pixels is then observed where a thresholding method is applied to cut off all the small blobs since they are usually noise. The remaining blobs that exceed the threshold will become the true white strips. A contour box for each true white strip, 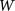 and 8-neighbourhood grey strips will be built around that strip as shown in Fig. 3.

However, not all white strips originate from the reflection regions. There are some that originate from the label as well as stains. Based on our initial assumption, the neighborhood information of a true white strip should be used to distinguish the plastic material. Thus, a likelihood test is utilized where all white strips will be verified by modeling the RGB color information of the strip probabilistically. 3D exponential distribution is used to model both the likelihood of true and false white strips. The true white strip, 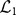 is modeled exponentially biased towards the white color or 255 pixel values of each 24-bit RGB color space. The false white strip, 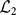 is modeled exponentially biased towards the black color or 0 pixel values of each 24-bit RGB color space. These operations are carried out for each individual pixel of the white strip, where the probability of each pixel will be integrated to derive the overall likelihood of each white strip. A likelihood ratio test is then performed to select the genuine white strips as indicated by 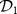. Let 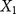 be the difference between the observed pixel RGB values and the white color while 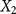 is the difference between the observed pixel RGB values and the black color. 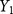 and 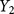 are the normalizing factors of each likelihood, while 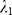 and 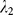 are the parameters dependent on the noise severity.
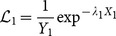
(1)

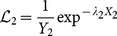
(2)

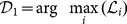
(3)


After the white strips have been verified, their neighborhood information is modeled probabilistically, mainly by analyzing the transmittance property. The main assumption in PET material detection is its white strips will usually have neighborhoods that are grey in color. These neighborhood boxes areas are called the grey strips. Besides, the detection is also supported by the distance of the grey strip to the white strip as well as the shape of the grey strip. For each white strip, maximum a posteriori method is employed to decide either the detected white strip is PET or non-PET. Several other applications have implemented a Bayesian approach in finding the best hypothesis for making a decision. Bayesian approach is popular in many applications, especially in video object tracking [20]. These include optimizing association in multiple object tracking used in [21] and in finding optimal threshold for color constancy used in [22]. The decisions on each white strip are accumulated in histogram form for the final classification. Let 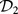 indicates the output of the maximum a posteriori where 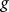 represents the grey strip prior of PET and non-PET material while 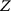 is the observed color information in the form of a RGB model.
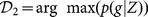
(4)


It can be reduced to equation 5 through Bayesian simplification.
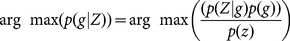
(5)


If we assume that 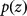 is equal for both PET and non-PET cases, equation 4 will be further simplified as
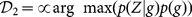
(6)


The grey strips of PET and non-PET are modeled by two prior information, which are 1) the distance between center of the grey strip and the white strip and 2) shape of the grey strip. The former parameter is a result of assuming that a closer neighborhood box to the white strip will better reflect the material identification. The latter parameter is obtained by assuming that a true reflection area will most likely have a slender shape. Besides, PET material is also more likely to have a narrow reflection area since most of the PET bottles are slim in shape. Let the prior parameters for distance and shape of the box be represented by 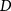 and 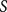 respectively.
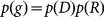
(7)


Exponential distribution is used to model directly the distance relationship between the grey strip and white strip. Similarly, the shape is also modelled by the exponential distribution based on the ratio value, 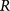, of width, 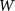 over height, 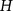 of the strip. The ratio will be capped by a threshold, 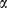 for the lower limit since the shape of the strip will be less significant if it is too slender.
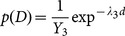
(8)

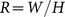
(9)

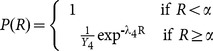
(10)


The priors are obtained even before the observations are sampled. Likelihood function is then employed to utilize the observations to distinguish either a particular white strip belongs to a PET or non-PET material. 3D Gaussian distribution is used to model the likelihood of detecting a PET material where the mean values, 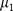 are biased towards grey color. This color is assumed if the pixel values of all three color channels are almost similar. For a non-PET material, the same Gaussian distribution is also used but the mean, 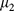 is biased towards non-grey color such that the values between R, G and B channels differ a lot. Both PET and non-PET likelihoods employ the same diagonal covariance matrix, 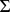.
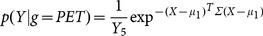
(11)

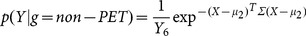
(12)


Thus, for any detected true white strip, they will be eight posterior probabilities calculated for each grey strip. The highest probability among all grey strips either it belongs to a PET or non-PET will be selected as the decision for that particular white strip. Hence, if the highest probability belongs to a PET material for grey strip number 4, the 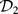 for that white strip will be a PET. A weightage, 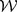 is then calculated based on the strip size.
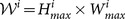
(13)where 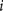 is the identifier for each detected white strip and 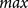 signifies the grey strip with the highest probability. These weightages of PET and non-PET materials is then accumulated in histogram form for the final decision, 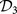. The decision that either the detected waste is a PET or non-PET material is decided collectively based on the likelihood test between the accumulated histogram values of PET and non-PET bins for all white strips 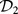. We define likelihood of a PET waste as 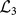, while likelihood of a non-PET waste is 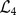.



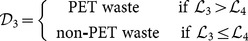
(14)A 1D Gaussian distribution is used to model the likelihood of both PET and non-PET waste. Supervised training is applied to find the optimal means and variances for both likelihoods. Let 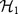 be the histogram bin of accumulated PET data while 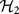 be the non-PET histogram representation. 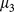 and 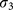 represent the mean and variance of 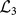 respectively while 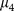 and 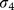 represent the mean and variance of 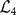 respectively. The supervised training is performed by taking some sample outputs of the histogram distribution of both PET and non-PET waste.
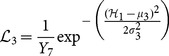
(15)

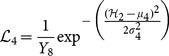
(16)where 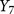 and 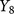 are the normalization parameters. Once the waste material is identified, a pneumatic or hydraulic mechanism is used to segregate the waste into the right collection bin. An accurate sorting system groups the same type of waste together so that the processed material will yield a high quality recyclable material.

## Results and Discussion

**Figure 4:**
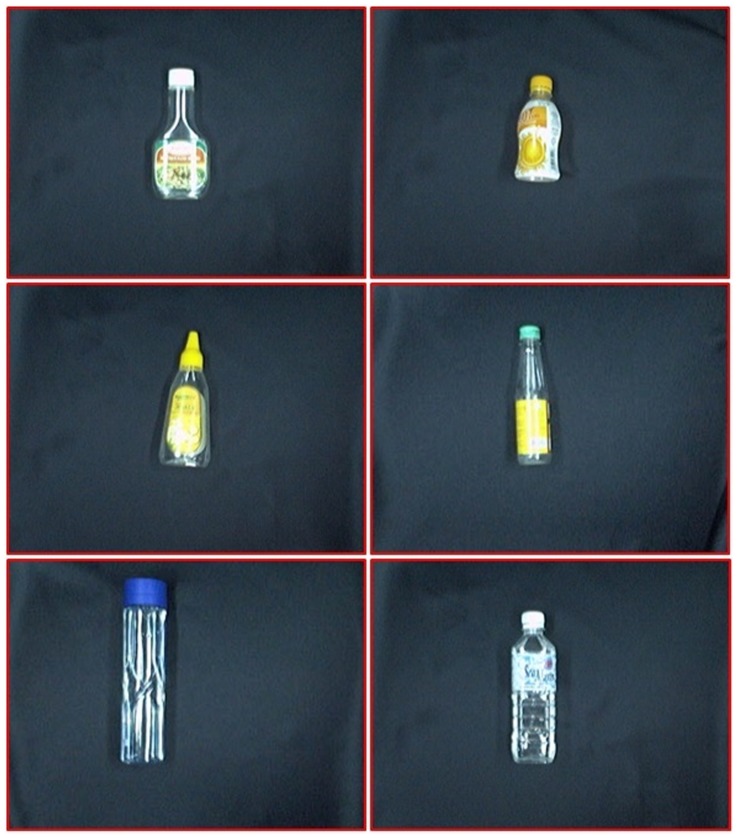
Samples of PET plastic waste.

**Figure 5:**
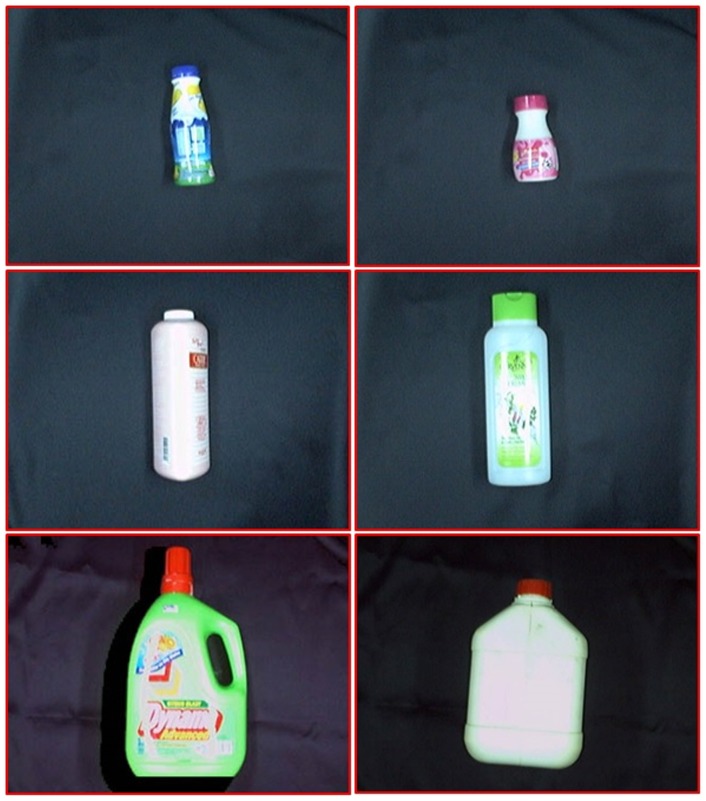
Samples of non-PET plastic waste.

Two groups of plastic waste were used to test the proposed system, which consisted of a combination of 300 PET and non-PET bottles. A CCD camera was used to capture the waste image with a frame size of 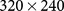 pixels. Out of 300 plastics wastes, 196 of them were made of non-PET material and the remaining 104 plastic wastes were made from PET material. Fig. 4 shows some examples of the PET plastic waste while Fig. 5 depicts some samples of non-PET plastic waste. The conveyor was fed with a waste item sequentially, which passed through an RGB camera before it was ejected to the right classification bin.

Most of the tested plastic wastes were not in perfect condition where dents and crumple were normal occurrences. Besides, most of the plastics still retained their label information, which covered a large part of the captured image. The size, shape and color of the bottles varied from one to another. The illumination condition also changed from frame to frame since some of the frames were affected by static shadows from nearby humans who happened to walk beside the machine. This situation can be observed in the video when the overall brightness of the image drops suddenly due to shadow occlusion. In our experiment, we considered a disjoint waste situation since our algorithm was not built to handle connected wastes. The captured data was then sent to a computer for real time image processing with an Intel Core2 Duo 2.4 GHz machine using OpenCV library.

The validation test was divided into two parts, where the first part employed a cross validation approach to verify the trained parameters are not a “one off”. The second part concerned the relative performance of the proposed method compared with three other benchmark methods. There were four performance metrics used to quantify the performance of the proposed system: precision (**P**), recall(**R**), accuracy (**A**) and error (**E**). Precision concerns how good is the system true detection relative to all detections while recall quantifies the true detection relative to ground truth detection. Accuracy is a measure of true classification while error is measure of false classification.

All four metrics have a range of 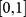 such that the system performs better if the value is closer to 1, except for error where the better system will register a lower error value. Let 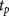 refer to true positive, 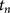 refer to true negative, 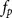 refer to false positive and 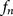 refer to false negative. The performance metrics were calculated as follows:
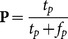
(17)

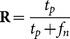
(18)

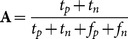
(19)

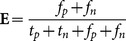
(20)


The first experimental component was a cross validation test of the proposed system where the plastic wastes were classified into four groups. Each group took a turn to be the training data while the remainder was tested based on the trained parameters. The purpose of the training was to determine the threshold value for the likelihood ratio test. Table 1 depicts the threshold value for each test group where the range is 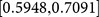, which were close to each other. This indicates that our test data was not biased towards any group and was randomly selected. The average threshold value was 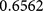 and the standard deviation was just 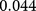. Thus, using the four sets of results, a good data set can be inferred if all results had similar performance regardless of which group was used to train the parameters. Table 2 summarizes the algorithm performance by performing a cross validation test using four performance metrics.

**Table 1:** Likelihood test threshold for each cross validation test group.

Data set	Group 1	Group 2	Group 3	Group 4
Likelihood test threshold	0.6366	0.6843	0.7091	0.5948

**Table 2:** Cross validation test on the proposed system.

Data set	Group 1	Group 2	Group 3	Group 4
Precision	0.7009	0.6863	0.7091	0.6832
Recall	0.7212	0.6731	0.7500	0.6635
Accuracy	0.7967	0.7800	0.8067	0.7767
Error	0.2033	0.2200	0.1933	0.2233

All four test groups returned more or less the same values for precision, recall, accuracy and error performance. The best precision was obtained by Group 3 with a precision value of 0.7091, while the best recall was also delivered by test Group 3 with a value of 0.7500. This was also reflected in accuracy and error performance where Group 3 once again delivered the most accurate detection with the least errors.

**Figure 6:**
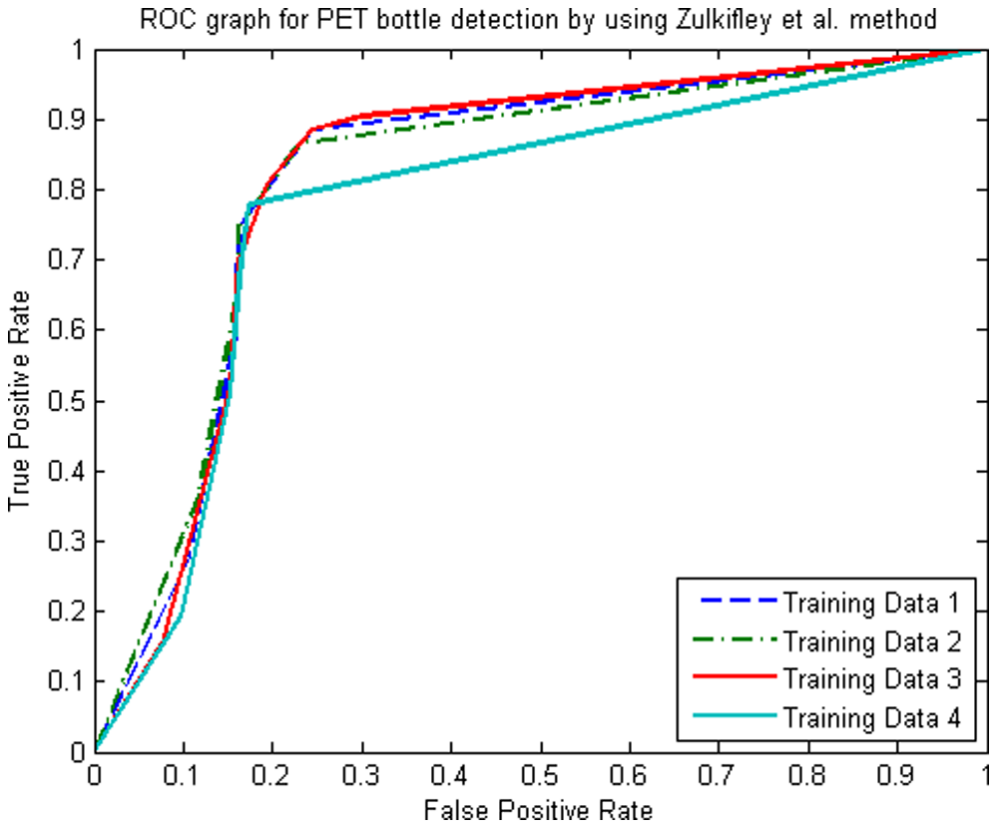
Receiver Operating Characteristic (ROC) curve the likelihood ratio test threshold: Cross validation of our proposed method.

On the other hand, the least accurate test group was Group 4 as it registered the worst performance in all four evaluation metrics. Hence, we could summarize that the optimal threshold value should be around 0.7000. The main reason for poor performance of Group 4 was due to unstable lighting illumination of the trained data. Fig. 6 shows the receiver operating characteristic (ROC) curves for all four test groups to analyze the sensitivity and specificity of the parameters. The curves indicate that the algorithm is moderately stable under variation of the likelihood test threshold. The working range is wide as any threshold value above 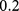 will produce a good detection system with no sudden drop in performance for all the tested range. Table 3 summarizes area under the curve (AUC) for all groups ROC curve. The AUC values for all four groups are relatively similar within the range of 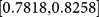.

**Table 3:** Area under the curve (AUC) for cross validation ROC curves of the method Zulkifley et al.

Method	Group 1	Group 2	Group 3	Group 4
AUC	0.7818	0.8258	0.8182	0.8206

For the second component, we benchmarked the proposed system that we denote as Zulkifley et al. against several recent algorithms in the literature. To focus on evaluating the quality of the extracted features of each method, we employed a common classifier for a fair comparison. The selected benchmark algorithms were as proposed by [14], [15], [23] and [18]. These four recent systems were built for real time application, which are similar to the proposed system. Table 4 summarizes the systems performance by using the same set of evaluation metrics as before.

**Table 4:** Performance comparison between the proposed method and the benchmark methods.

Method	Precision	Recall	Accuracy	Error
Zulkifley et al.	0.6952	0.7019	0.7900	0.2100
House et al.	0.0957	0.0865	0.4000	0.6000
Shahbudin et al.	0.3614	0.2885	0.5767	0.4233
Wahab et al.	0.5918	0.2788	0.6833	0.3167
Ramli et al.	0.5223	0.7884	0.6767	0.3233

Based on the experimental results, the proposed algorithm obtained the best precision index, which is 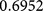 and relatively good measures for recall, accuracy and error. The main reason for better classification results can be attributed to the integrated approach of color, size and shape information in decision making. The method by Wahab et al. performed the second best in the precision metric while method by Shahbudin et al. performed the third best for recall evaluation. Both methods by Wahab et al. and Shahbudin et al. relied on shape information alone. They utilized the intensity channel to build the silhouette of the waste shape. However, the method by Wahab et al. performed a background subtraction method first to extract the foreground object. This step allowed them to identify the size of the plastic waste accurately. Unlike method by Shahbudin et al. that relies on edge detection alone, the detected edges were affected by the conveyor line information. Hence, the method by Wahab performs better in term of precision but less accurate for recall evaluation. Method by Ramli et al. returned the highest recall value with 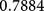, which is slightly higher than method by Zulkifley et al. with 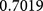. They managed to obtain good recall value because of the nature of their feature extraction that emphasizes on less false detection rather miss detection. The downside of Ramli et al. approach can be observed through high error rate since the threshold balance tip towards false detection which lead to high miss detection rate.

Another advantage of the method by Zulkifley et al. over the methods by Wahab et al., Shahbudin et al. and Ramli et al. is that it does not require the plastic waste to be in perfect shape as it is able to identify the material even when bent or crushed. The dependence on shape information alone reduced the classification accuracy of the methods by Wahab et al. and Shahbudin et al. when the plastic waste was crushed. The enhanced structuring element approach by Ramli et al. tried to accommodate for imperfection in detected shape, but still it just has larger tolerance. The worst performing method was by House et al. with the lowest precision and accuracy values since they just relied on grey scale histograms of the plastic waste appearance. The reasons relate to it may have big label information as its surface may also be tainted by some contaminant. The detection accuracy would degrade even before factoring in the case of illumination change as the test data set did contain some lighting variation. Hence, grey scale information alone was not a good indicator to detect a PET material.

**Figure 7:**
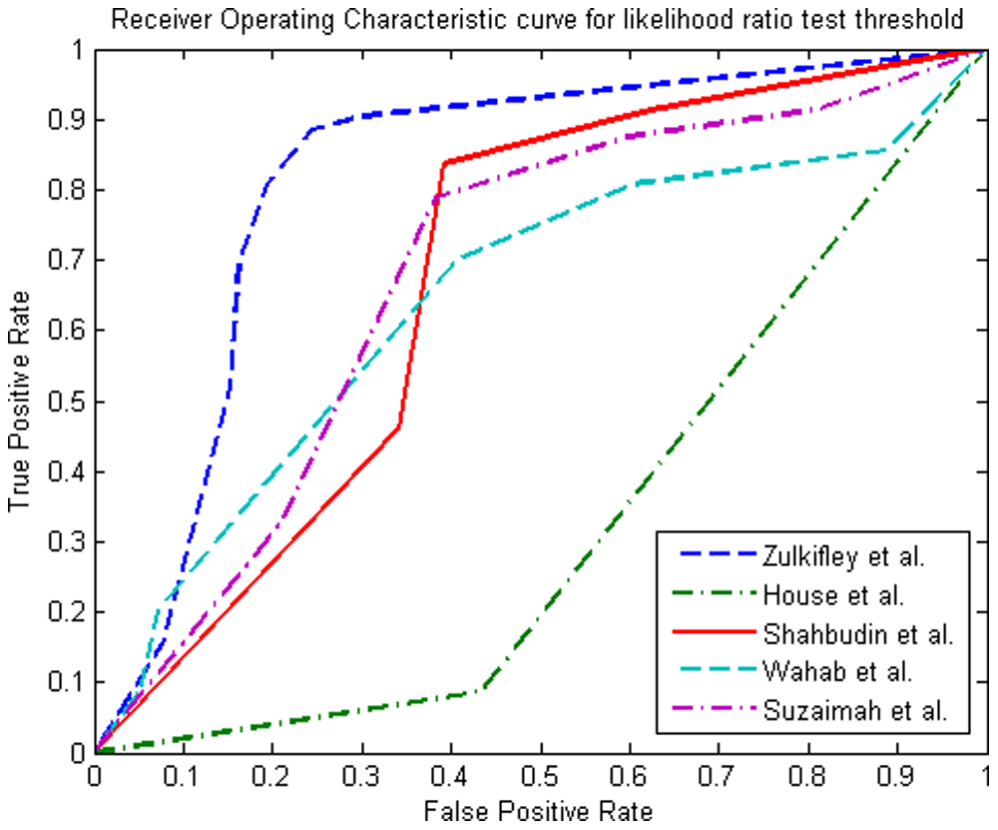
Receiver Operating Characteristic (ROC) curve for threshold of likelihood ratio test: Benchmarks comparison.

Fig. 7 shows the ROC curve for all benchmark algorithms. The method by Zulkifley et al. performed the best except for the region 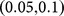, where the method by Wahab et al. performed slightly better. The curve indicates that our method was the least affected by the threshold change of the likelihood ratio test. Interestingly, for a threshold of less than 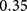, the method by Wahab et al. had a better sensitivity and specificity properties compared to the method by Shahbudin et al. On the flip side, the method by Shahbudin et al. outperformed the method by Wahab et al. when the threshold value was more than 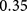. The main reason was the edge detection used in Shahbudin et al. required a certain minimum intensity to function properly. A low threshold made it less sensitive as it could not accurately distinguished the edge. The performance by Ramli et al. is worse than Wahab et al. for false positive rate less than 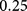, while it performs worse than Shahbudin et al. for false positive rate bigger than 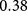. The method by House et al. had the most unstable detection when the threshold for the likelihood ratio test was varied. The ratio between PET and non-PET for the method by House et al. worked the best if it was close to 1, which indicated there was not much difference between grey level information of a PET and non-PET bottle. Hence, the most stable method under threshold variation was Zulkifley et al., followed by Ramli et al., Shahbudin et al., Wahab et al. and House et al. based on the AUC values in Table 5. AUC of Zulkifley et al. is also significantly higher than the benchmarked algorithms with 0.8235 compared to the rest with AUC value of less than 0.7.

**Table 5:** Area under the curve (AUC) for the benchmarked algorithms.

Method	Zulkifley et al.	House et al.	Shahbudin et al.	Wahab et al.	Ramli et al.
AUC	0.8235	0.3301	0.6788	0.6469	0.6847

## Conclusions

The proposed system managed to process the plastic waste in real time. The main novelty of the system was its probabilistic approach to model the color, size and distance of the neighborhood strip to distinguish between PET and non-PET material. The system achieved the classification accuracy of 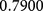 with a low error of 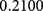. Precision and recall values of the algorithm were 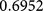 and 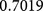 respectively. The algorithm was able to classify PET and non-PET waste with a high accuracy even though the lighting condition was not stable and the waste appearance was contaminated with stains as well as large label information. In the future, the system could be further improved by using an integrated approach in modeling the neighborhood information instead of using the best region only. The system could also be implemented in general purpose graphics processing unit such as a CUDA platform for faster processing.
